# Randomised controlled trial of a theory-based behavioural intervention to reduce formula milk intake

**DOI:** 10.1136/archdischild-2018-314784

**Published:** 2018-05-14

**Authors:** Rajalakshmi Lakshman, Stephen J Sharp, Fiona Whittle, Annie Schiff, Wendy Hardeman, Lisa Irvine, Ed Wilson, Simon J Griffin, Ken K Ong

**Affiliations:** 1 MRC Epidemiology Unit and UKCRC Centre of Excellence in Diet and Activity Research (CEDAR), University of Cambridge, Cambridge, UK; 2 Health Promotion Research Group, School of Health Sciences, University of East Anglia, Norwich, UK; 3 Norwich Medical School, University of East Anglia, Norwich, UK; 4 Cambridge Centre for Health Services Research, Department of Public Health and Primary Care, University of Cambridge, Cambridge, UK; 5 Department of Paediatrics, University of Cambridge, Cambridge, UK; 6 Primary Care Research Unit, Institute of Public Health, School of Clinical Medicine, University of Cambridge, Cambridge, UK

**Keywords:** obesity, nutrition

## Abstract

**Objective:**

To assess the efficacy of a theory-based behavioural intervention to prevent rapid weight gain in formula milk-fed infants.

**Design:**

In this single (assessor) blind, randomised controlled trial, 669 healthy full-term infants receiving formula milk within 14 weeks of birth were individually randomised to intervention (n=340) or attention-matched control (n=329) groups. The intervention aimed to reduce formula milk intakes, and promote responsive feeding and growth monitoring to prevent rapid weight gain (≥+0.67 SD scores (SDS)). It was delivered to mothers by trained facilitators up to infant age 6 months through three face-to-face contacts, two telephone contacts and written materials.

**Results:**

Retention was 93% (622) at 6 months, 88% (586) at 12 months and 94% attended ≥4/5 sessions. The intervention strengthened maternal attitudes to following infant feeding recommendations, reduced reported milk intakes at ages 3 (−14%; intervention vs control infants), 4 (−12%), 5 (−9%) and 6 (−7%) months, slowed initial infant weight gain from baseline to 6 months (mean change 0.32 vs 0.42 SDS, baseline-adjusted difference (intervention vs control) −0.08 (95% CI −0.17 to −0.004) SDS), but had no effect on the primary outcome of weight gain to 12 months (baseline-adjusted difference −0.04 (−0.17, 0.10) SDS). By 12 months, 40.3% of infants in the intervention group and 45.9% in the control group showed rapid weight gain (OR 0.84, 95% CI 0.59 to 1.17).

**Conclusions:**

Despite reducing milk intakes and initial weight gain, the intervention did not alter the high prevalence of rapid weight gain to age 12 months suggesting the need for sustained intervention.

**Trial registration number:**

ISRCTN20814693.

**What is already known on this topic?**

- Rapid weight gain during infancy is consistently associated with later obesity, hence infancy could be a critical period for obesity prevention.
- Formula milk-fed infants grow faster than breastfed infants, and energy intakes of formula milk-fed infants predict weight gain and childhood body mass index.
- Although most infants are fed formula milk, no study has effectively reduced intakes among formula milk-fed infants.

**What this study adds?**

- This behavioural intervention reduced milk intakes and slowed initial weight gain to age 6 months, but not weight gain to 12 months.
- Infants in both groups consumed substantially higher energy than recommended and almost half showed rapid weight gain (upwards crossing >1 centile band) in the first year.
- Interventions to avoid rapid weight gain in infancy need to be sustained and scalable.

## Introduction

Evidence from observational studies supports the robust and highly consistent association between rapid weight gain during infancy and later obesity^1^ and also with cardiovascular disease risk factors.^2^ In the most recent systematic review, 45/46 studies reported a positive association between infancy weight or weight gain and later childhood overweight.^3^ Infancy is a period of rapid growth, habit formation and developmental plasticity,^4^ hence it is recommended by the World Health Organization as an important time to target obesity prevention.^5^

Energy deposition as a percentage of total energy requirements decreases from 40% at age 1 month to 1%–2% from 12 months until mid-adolescence.^6^ Therefore, weight gain during infancy is more closely related to energy intake than is weight gain in childhood or in later life. In 2004, the WHO and other international bodies reduced the estimated average energy requirements (EAR) for infants by 15%–20% and UK dietary reference values for energy were similarly revised in 2011.^6^ However, there is wide interindividual variation among formula milk-fed infants in their energy intakes, which are positively associated with rate of infancy weight gain and childhood body mass index (BMI).^7^ Although the benefits of breast feeding are well recognised, only 23% of UK infants are exclusively breast fed at age 6 weeks.^8^ Hence, alongside breast feeding promotion, optimising the diet and growth of formula milk-fed infants may contribute to reducing the prevalence of childhood obesity.

Systematic reviews of early life interventions to prevent childhood obesity found that research in this area is recent and evolving.^9 10^ The latest review in 2016 found that of 26 interventions, 7 of the 18 behavioural interventions and two of the eight biologic interventions were effective.^9^

We aimed to evaluate the efficacy, mechanisms and cost of a theory-based, behavioural intervention to reduce formula milk intake and prevent excess weight gain during infancy in an explanatory, single (assessor) blind, parallel group, individually randomised controlled trial (RCT) of parents (usually mothers) and their infants.

## Methods

### Participants

The full trial protocol has been published elsewhere.^11^ Healthy, full-term infants receiving formula milk within 14 weeks of birth were eligible to participate. Exclusion criteria were: low birth weight (<2500 g), preterm (<37 weeks’ gestation), receiving special formulas (soya-based, lactose-free, hydrolysed or antireflux formulas), major malformations and hormonal or metabolic diseases which might interfere with nutrition or growth. Participants were identified by general practitioners (42%), research staff on a postnatal hospital ward (23%), via a mail-out using the centralised National Health Service (NHS) integrated database ‘SystmOne’ (27%), or self-referred.

### Intervention

Intervention development has been previously described.^12^ It followed an iterative process and included systematic reviews of the literature^13–15^ and qualitative studies.^16^ The intervention included three components: a motivational component (based on social cognitive theory),^17^ an action planning component to help translate motivation into action (including goal setting and self-monitoring) and a coping planning component helping parents to deal with difficult situations by making ‘if…then…’ plans (the latter two components were based on ‘implementation intentions’ (online supplementary figure 1)).^18^ The aims of the intervention were to reduce formula milk intake (in line with 2004 WHO EAR for energy),^6^ and to promote responsive feeding and monitor growth to prevent excess weight gain (crossing upwards centile bands on growth charts >±0.67 SD score (SDS)). The intervention encouraged mothers to recognise infants’ satiety cues, not to force infants to finish the bottle, recognise that crying was not always due to hunger (infants may be thirsty or tired) and not to feed the infants every time they cried (try water or a dummy). It was delivered by trained facilitators (research nurses) to mothers of infants up to 6 months of age through three 30–45 min face-to-face sessions (at baseline and ages 4 and 6 months) and two 15–20 min telephone contacts (ages 3 and 5 months) in addition to two leaflets and stickers (with the new recommendations) to put on formula milk powder tins. We selected behaviour change techniques (BCT) with evidence of effectiveness in changing dietary behaviours,^19^ to target the hypothesised theory-based mediators of our intervention. We used Abraham and Michie’s taxonomy^20^ to define the BCTs and operationalise them as intervention strategies in the intervention protocols (online supplementary table 1). The attention control group mothers received the same number of contacts during which facilitators discussed general topics including other aspects of formula milk feeding (online supplementary table 2).

10.1136/archdischild-2018-314784.supp1 Supplementary data

10.1136/archdischild-2018-314784.supp2 Supplementary data

### Outcomes

The primary outcome was change in weight SDS from birth to 12 months. Since this was an explanatory RCT and we aimed to contribute to the sparse literature on the behavioural mechanisms of the development of childhood obesity,^21^ we measured a number of factors along the casual pathway (online supplementary figure 2).

10.1136/archdischild-2018-314784.supp3 Supplementary data

Anthropometry data were collected by trained research assistants blinded to group allocation using standard operating procedures at baseline, age 6 and 12 months. Infant weight, length, BMI, and abdominal and head circumference were converted to SDS adjusted for age and sex based on the 2006 WHO Growth Standard which describes the optimal growth of healthy, breastfed children.^22^ Rapid/excess weight gain was defined as crossing ≥+0.67 SDS (one centile band). Implausible values were excluded (beyond ±6 SDS).

Data about the hypothesised mediators of behaviour change (maternal attitudes, self-efficacy, outcome expectancies and intentions with regard to following feeding recommendations) were collected using a validated questionnaire.^23^

Questionnaires at each contact (baseline, and infant’s ages 3, 4, 5 and 6 months) assessed total milk intake, number of solid feeds and age at introduction of solids.^23^ Detailed diet data were collected at 8 months using a 4-day diet diary. The diet diary was analysed using the Diet in Nutrients Out computer package^24^ by the dietary assessment team at the Medical Research Council Human Nutrition Research Unit, blinded to group allocation. Health service utilisation and maternal quality of life data were collected at 6 and 12 months.^11^

### Statistical analysis

The sample size was estimated based on a predicted difference of 0.20–0.21 SDS in weight change from birth to 12 months assuming a 10%–15% reduction in milk intake between the intervention and control groups.^7^ Allowing for a 15% loss to follow-up, 300–350 infants in each group would provide 80% power at a two-sided 5% significance level to detect this difference.^11^ Central telephone randomisation was based on a computer-generated randomisation list. All data were double-entered and cleaned by staff blinded to group allocation.

The primary efficacy outcome, change (from birth to age 12 months) in weight SDS, was analysed using linear regression with birth weight SDS and a randomised group indicator variable as covariates. The missing indicator method^25^ was used so that infants with missing values of a variable at birth/baseline were included in the analysis. A similar method was used for other continuous secondary outcomes. For the primary outcome, the interaction between randomised group and formula feeding status (fully/partially formula fed) was tested by including the interaction parameter in the regression model. The ‘per protocol’ analysis included participants completing the intervention programme based on attendance at 4/5 sessions (80% attendance). The binary outcomes ‘excess weight gain/loss’ (±0.67 SDS change from baseline) were analysed using logistic regression, with baseline weight SDS and a randomised group indicator variable as covariates. The analysis was performed using Stata V.14.^26^

A within-trial cost-consequences analysis from the perspective of the UK NHS comparing the *Baby Milk* intervention with control was conducted. Unit costs were obtained from national sources including Personal Social Services Research Unit costs and NHS reference costs.^27 28^

## Results

### Baseline characteristics

**Figure 1:**
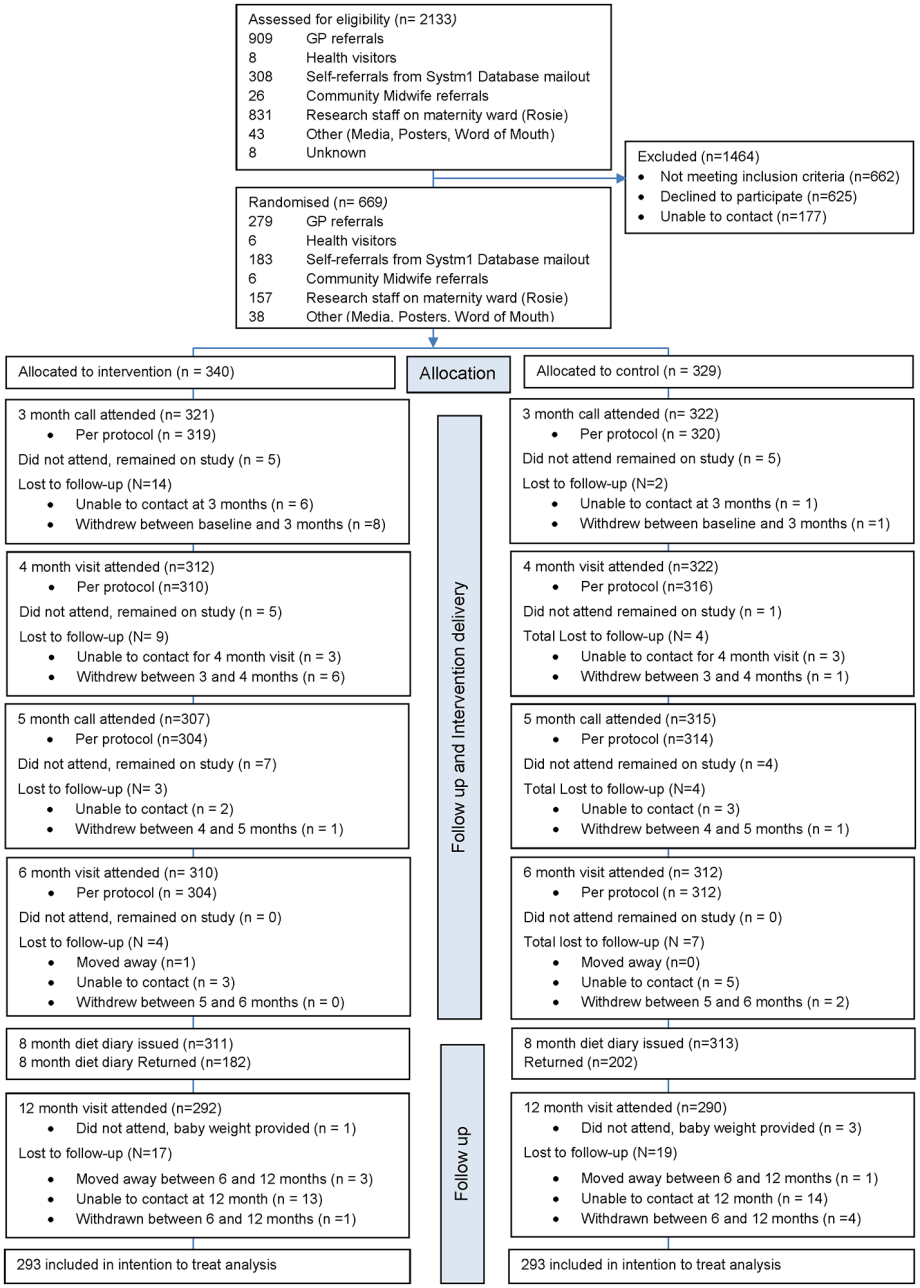
Trial profile. GP, general practitioner.

Between March 2011 and June 2015, 669 infants were randomised (340 intervention, 329 control) of the 2133 assessed for eligibility (31%). We assessed 622 infants (93%; 310 intervention, 312 control) at 6 months, and 586 infants (88%; 293 intervention, 293 control) at 12 months and this formed the intention-to-treat population (figure 1). Engagement was high in both intervention and control groups (94%; 308 intervention, 319 control attended ≥4/5 sessions) and 580 infants were included in the per-protocol population (87%; 288 intervention, 292 control).

The mean (SD) age of the infants at baseline was 2.3 (1.0) months; gestational age was 39.6 (2.0) weeks, indicating full term; birth weight was 3.4 (0.5) kg and weight at baseline 5.5 (0.9) kg. Of the 669 infants, 46% were female, 94.4% fully formula milk fed at 6 months and 52.5% first born. The mean (SD) age of the mothers was 31.6 (5.8) years, BMI was 27.9 (5.4) kg/m^2^ and weight gain during pregnancy was 12.9 (6.8) kg. There were no differences in baseline characteristics among participants who completed the trial and those who were randomised (table 1).

**Table 1:** Baseline characteristics of participants who started and completed the trial

	Enrolled		Completed 12-month FU	
	Control (n=329)	Intervention (n=340)	Control (n=292)	Intervention (n=293)
**Infants**				
Age (months)	2.3 (1.0)	2.3 (1.0)	2.3 (0.8)	2.3 (1.1)
Gestational age (weeks)	39.6 (2.7)	39.7 (1.4)	39.5 (2.8)	39.7 (1.4)
Female (%)	150 (45.6)	158 (46.5)	131 (44.9)	137 (46.8)
Fully formula fed at 6 months (%)	277 (93.3)	277 (95.5)	262 (93.9)	264 (95.7)
First born (%)	167 (53.0)	174 (51.9)	167 (53.0)	174 (51.9)
Birth weight (kg)	3.41 (0.5)	3.47 (0.5)	3.41 (0.5)	3.45 (0.5)
Birth weight SDS	0.22 (1.0)	0.31 (0.9)	0.20 (1.0)	0.27 (0.9)
Weight SDS	−0.16 (0.9)	−0.06 (0.9)	−0.16 (0.9)	−0.11 (0.9)
BMI SDS	−0.11 (0.9)	−0.03 (0.9)	−0.11 (0.9)	−0.08 (0.9)
Length (cm)	58.3 (3.1)	58.5 (3.3)	58.4 (3.1)	58.5 (3.3)
Formula milk intake (mL/day)	898.1 (219.7)	895.9 (217.6)		
**Mothers**				
Age (years)	31.3 (5.8)	31.9 (5.9)	31.4 (5.5)	32.2 (5.7)
BMI (kg/m^2^)	27.8 (5.4)	28.1 (5.5)	27.8 (5.5)	28.2 (5.4)
Pregnancy weight gain (kg)	12.7 (6.9)	13.0 (6.8)	12.9 (6.8)	13.1 (6.7)
Age completed education (years)	19.5 (3.6)	19.6 (3.5)	19.6 (3.2)	19.6 (3.4)
Degree or higher	120 (38.3%)	123 (37.4%)	110 (39.1%)	113 (39.8%)
A-level, below degree	70 (22.4%)	72 (21.9%)	66 (23.5%)	63 (22.2%)
GCSE/vocational	118 (37.7%)	128 (38.9%)	102 (36.3%)	103 (36.3%)
Below GCSE or no formal qualifications	5 (1.6%)	6 (1.8%)	3 (1.1%)	5 (1.8%)
Occupation				
Professional, higher managerial, administrative	158 (52.3%)	142 (43.3%)	143 (52.4%)	128 (44.9%)
Lower managerial, intermediate	93 (30.8%)	125 (38.1%)	84 (30.8%)	110 (38.6%)
Technical, semiroutine, routine	39 (12.9%)	54 (16.5%)	35 (12.8%)	43 (15.1%)
Never employed	12 (4.0%)	7 (2.1%)	11 (4.0%)	4 (1.4%)
White ethnicity	295 (93.1%)	322 (95.8%)	266 (93.3%)	279 (96.5%)
Married	184 (58.2%)	190 (56.9%)	166 (58.2%)	172 (59.7%)
Smoked during pregnancy	38 (12.0%)	38 (11.3%)	31 (10.9%)	28 (9.7%)
Consumed alcohol during pregnancy	64 (20.2%)	58 (17.2%)	55 (19.3%)	52 (17.9%)

Means (SD) for continuous variables, numbers (%) for categorical variables; SDS calculated using WHO 2006 growth charts.

BMI, body mass index; FU, follow-up; GCSE, General Certificate of Secondary Education; SDS, SD score.

### Weight gain

**Figure 2:**
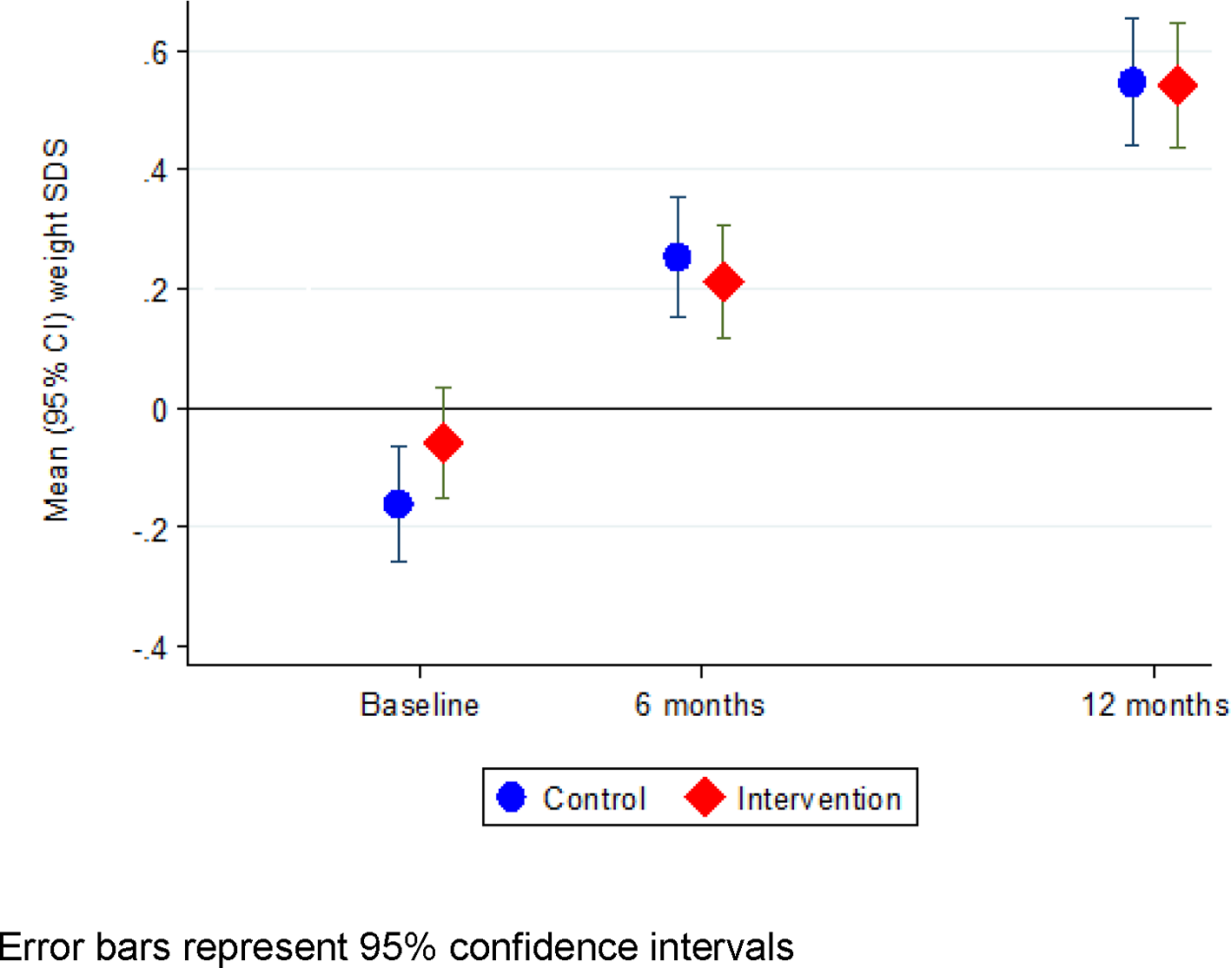
Weight SDS (WHO 2006 standard) in intervention and control group participants. SDS, SD score.

Table 2 shows the between-group differences in changes in anthropometry and safety outcomes from birth/baseline to ages 6 and 12 months. The mean (SD) change in weight SDS from baseline to 6 months was 0.32 (0.55) in the intervention group and 0.42 (0.53) in the control group, a baseline-adjusted difference of −0.08 (95% CI −0.17 to −0.004) (figure 2). The mean (SD) change in weight SDS from birth to 12 months was 0.28 (0.96) in the intervention group and 0.35 (1.05) in the control group, representing a difference (adjusted for birth weight SDS) of −0.04 (95% CI −0.17 to 0.10, p=0.61). Results were similar in the per-protocol population. There was no interaction between the intervention and formula feeding (fully vs partially formula fed), p=0.38.

**Table 2:** Between-group differences in change in anthropometry and safety outcomes from birth/baseline to ages 6 and 12 months

	6 months			12 months		
	Control	Intervention	Difference (95% CI) Intervention versus control	Control	Intervention	Difference (95% CI) Intervention versus control
**Primary outcome**						
Change in weight SDS from birth	0.05 (1.0)	−0.05 (0.91)	−0.06 (−0.19 to 0.06)	0.35 (1.05)	0.28 (0.96)	−0.04 (−0.17 to 0.10)
**Secondary outcomes**						
Rapid weight gain ≥+0.67 SDS (%)	94 (28.6%)	74 (21.8%)	OR: 0.74 (0.51 to 1.07)	151 (45.9%)	137 (40.3%)	OR: 0.84 (0.59 to 1.17)
Change in weight SDS from baseline	0.42 (0.53)	0.32 (0.65)	**−0.08 (−0.17 to −0.004)**	0.70 (0.70)	0.65 (0.72)	−0.04 (−0.14 to 0.07)
Change in BMI SDS from baseline	0.34 (0.69)	0.27 (0.73)	−0.07 (−0.17 to 0.04)	0.69 (0.89)	0.68 (0.86)	−0.01 (−0.14 to 0.12)
Change in abdominal circumference SDS from baseline	3.96 (2.8)	3.59 (2.8)	−0.33 (−0.70 to 0.05)	5.95 (3.1)	5.68 (3.1)	−0.27 (−0.70 to 0.16)
Sum of skinfold thickness (mm)				16.4 (3.1)	16.5 (3.0)	0.10 (−0.42 to 0.61)
Abdominal ultrasound subcutaneous fat thickness (cm)				0.46 (0.12)	0.46 (0.12)	0.00 (−0.02 to 0.03)
Abdominal ultrasound visceral fat thickness (cm)				2.5 (0.5)	2.6 (0.5)	0.07 (−0.02 to 0.16)
Change in mother’s weight from baseline (kg)	−1.34 (3.7)	−1.25 (3.4)	0.03 (−0.62 to 0.68)	−2.99 (5.2)	−2.82 (5.6)	0.12 (−0.83 to 1.08)
Change in mother’s BMI from baseline (kg/m^2^)	−0.50 (1.3)	−0.46 (1.9)	−0.00 (−0.23 to 0.24)	−1.10 (1.9)	−1.04 (2.1)	0.02 (−0.33 to 0.37)
**Safety outcomes**						
Drop in one centile band <0.67 SDS (%)	7 (2.1%)	7 (2.1%)	OR: 1.08 (0.36 to 3.22)	7 (2.1%)	8 (2.4%)	OR: 1.20 (0.42 to 3.44)
Change in length (cm) from baseline	8.97 (2.7)	8.71 (2.7)	−0.22 (−0.53 to 0.09)	17.38 (2.3)	17.19 (3)	−0.17 (−0.56 to 0.21)
Change in head circumference SDS from baseline	0.19 (0.49)	0.13 (0.50)	−0.06 (−0.14 to 0.02)	0.24 (0.60)	0.18 (0.64)	−0.06 (−0.15 to 0.04)
Maternal safety outcomes*						
Change in SF-8 mental health score from baseline	1.0 (8.3)	0.4 (8.2)	−0.60 (−1.77 to 0.58)	1.2 (8.0)	−1.1 (9.7)	**−2.29 (−3.60 to −0.99)**
Change in SF-8 physical health score from baseline	2.4 (8.8)	3.1 (9.0)	0.07 (−1.05 to 1.19)	2.6 (8.3)	2.9 (9.7)	−0.11 (−1.26 to 1.05)
Change in health VAS score from baseline	1.7 (14.4)	2.3 (16.2)	−0.64 (−2.8 to 1.57)	0.8 (15.3)	0.5 (19.6)	−1.03 (−3.68 to 1.62)
Change in anxiety score from baseline	−1.5 (9.1)	0.5 (11.5)	1.32 (−0.22 to 2.85)			

Mean (SD) for continuous variables, number (%) for categorical variables; SDS calculated using WHO 2006 growth charts. Differences are adjusted for value of outcome at birth/baseline if this was measured. Values displayed in bold font are statistically significant (P<0.05).

*Mother’s quality of life was assessed using the SF-8 and EuroQoL VAS. Maternal anxiety was measured using Spiegelberger Short State Anxiety Inventory.

BMI, body mass index; SDS, SD score; SF, Standard Form; VAS, visual analogue scale.

At 6 months, 21.8% of infants in the intervention group compared with 28.6% in the control group (OR 0.74, 95% CI 0.51 to 1.07) gained rapid weight (≥+0.67 SDS increase from baseline). At 12 months, these proportions were 40.3% in the intervention group versus 45.9% in the control group (OR 0.84, 95% CI 0.59 to 1.17) (online supplementary figure 3).

10.1136/archdischild-2018-314784.supp4 Supplementary data

### Reported milk intakes

**Figure 3:**
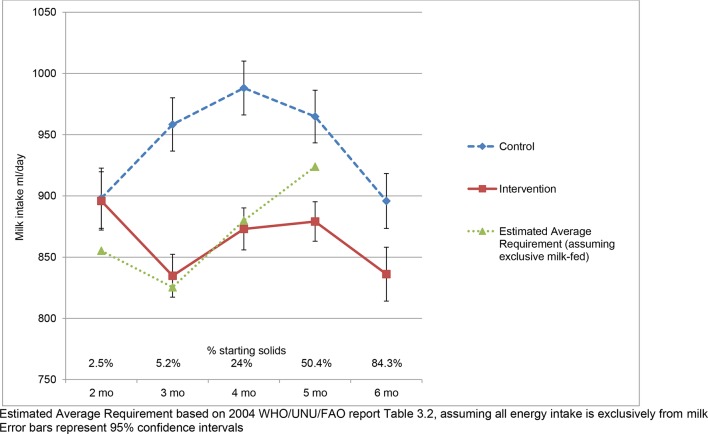
Milk intake (mL/day) in intervention and control group infants.

The average daily reported total milk intake at baseline was 897 mL/day, which is 5% more than the WHO EAR (855 mL/day, presuming all energy is from milk). The intervention was effective in reducing milk intake (the target behaviour) at ages 3 months (mean reduction, intervention vs control: 123.5 (95% CI 95.5 to 151.6) mL/day), 4 months (115.1 (95% CI 87.1 to 143.0) mL/day), 5 months (85.7 (95% CI 58.8 to 112.6) mL/day) and 6 months (59.7 (95% CI 28.3 to 91.1) mL/day) (figure 3, online supplementary table 3). This equated to a difference in milk intake between intervention and control groups of −14%, −12%, −9% and −7% at 3, 4, 5 and 6 months, respectively. The mean (SD) age for introduction of solid feeds was 4.9 (0.84) months and over half the infants were consuming solids before the recommended age of 6 months (2.5% at 2, 5.2% at 3, 24% at 4, 50.4% at 5, and 84.3% at 6 months) with no differences between groups. There was no difference between groups in the reported number of solid feeds at ages 2, 3, 4, 5 and 6 months. Average reported energy intake at age 8 months was similar in both groups (770.1 kcal/day vs 776.4 kcal/day, intervention vs control) and 16% higher than the 2004 WHO EAR (666 kcal/day at age 7–9 months).^6^

### Maternal attitudes

At the infant’s age 6 months, the intervention increased mothers’ confidence (self-efficacy) to follow the feeding recommendations in difficult situations, increased their ‘expected benefits’ (outcome expectancy) of following the recommendations and increased their intentions to follow the recommendations. No between-group difference was found in changes from baseline in mothers’ confidence to follow the feeding recommendations without partner/family support (online supplementary figure 4).

10.1136/archdischild-2018-314784.supp5 Supplementary data

At infant’s age 6 months, the intervention increased mothers’ ‘*worry about the baby gaining too much weight*’, increased ‘*thinking it was possible to feed the baby too much*’ and increased mothers’ ‘*confidence that they could feed their baby so the baby did not gain too much weight*’ (online supplementary figure 5).

10.1136/archdischild-2018-314784.supp6 Supplementary data

### Intervention costs

The cost of delivering the intervention and control group protocols was estimated to be £323 and £260 per infant, respectively. The number of reported healthcare contacts was low in both groups, and there was no difference in healthcare use or costs (online supplementary tables 4 and 5).

## Discussion

This is the first trial of any behavioural intervention to avoid excessive energy intakes among formula milk-fed infants. The intervention reduced reported milk intakes at ages 3, 4, 5 and 6 months, and slowed initial weight gain to age 6 months. However, the effect of the intervention on weight gain was not sustained to age 12 months, the primary outcome. At age 8 months, infants in both groups consumed on average >100 kcal/day more total energy than their estimated average requirement.

Since almost three-quarters (73%) of infants in the UK receive formula milk by age 6 weeks, with this proportion rising to 8 in 10 (83%) by 4 months and nearly 9 in 10 (88%) by 6 months,^8^ it is important that when mothers choose to feed their infants formula milk they are supported to feed their infants appropriately. We found that infants were reported to consume 5% more formula milk than recommended at the start of the study (average age 2.3 months). Past attempts to reduce energy intake among formula milk-fed infants have had limited success. One trial of an educational intervention to promote responsive formula milk feeding (recognise satiety cues) did not find any difference in mean formula milk intakes at ages 4–5 months (which were >1100 mL/day in both groups) and weight gain was greater in the intervention group than in the control group (p<0.01).^29^ Similarly, a trial comparing different types of bottle design found no significant differences in anthropometry at ages 2, 3 and 4 months.^30^

Despite the high prevalence of obesity already by the age at school entry, most prevention efforts have focused on school-age children and adolescents and have had limited success.^31^ Studies evaluating early life interventions during pregnancy and the first 2 years of life are recent.^9 10^ The latest systematic review (in 2016) identified 26 interventions of which nine reported a favourable effect on slower growth (7/18 behavioural, 2/8 hydrolysed/lower protein formula).^9^ Of these, the seven effective behavioural interventions targeted maternal/family and child, sleep, diet and physical activity, and their intervention durations were invariably longer than the *Baby Milk* intervention (online supplementary table 6). The *Baby Milk* intervention adds to this body of evidence by showing that a behavioural intervention targeting formula milk intake can also have an initial favourable effect on slower weight gain when milk is the main diet. None of the behavioural interventions have shown long-term effectiveness beyond the duration of the intervention and unfortunately the *Baby Milk* intervention was similarly ineffective at sustaining changes beyond the intervention period. There was no difference between the two groups in reported energy intakes at 8 months and weight gain to 12 months supporting the chronic disease model for the prevention and treatment of obesity which suggests that sustained intervention may be required.^32^

A limitation of the trial is that participants were mainly white (95%) although education levels were similar to the UK population (38% had a degree or higher qualification compared to 40% of 25-40 year olds in England and Wales).^33^ This could be due to the geography where recruitment took place and the motivation of mothers to take part in research with consequences for external validity and generalisability, which is not unique to our trial but a challenge for most RCTs.

## Conclusions

The high prevalence of excessive energy intakes and rapid weight gain in the *Baby Milk* trial highlight the importance of interventions starting in early life. Consistent with other UK data,^34^ we found that average reported energy intake at age 8 months (773 kcal/day) was substantially higher than the WHO estimated average requirement (666 kcal/day). Almost half (43%) showed rapid weight gain in the first year, making this a priority area for further intervention and behaviour change. The lack of effectiveness on reported energy intakes and weight gain beyond the duration of the *Baby Milk* intervention suggests that future interventions need to provide sustained support that is adaptive to the changes with infant age in feeding practices and context, and may need to target other dietary behaviours, physical activity and possibly sleep. This could be feasible if supported by digital technologies, such as text messaging, mobile applications and websites. Careful development work and feasibility testing will be required to ensure that such support is based on theory and evidence, meets the needs and preferences of the target group and includes strategies to enhance initial and sustained engagement.^35^ Furthermore, there is a need for interventions to promote consistent and appropriate social and professional norms about a healthy pattern of growth and infant feeding.^36^
