# Supplementary material for: Randomised controlled trial of a theory-based behavioural intervention to reduce formula milk intake
Source: Arch Dis Child. 2018 May 14;103(11):1054–60. doi: 10.1136/archdischild-2018-314784 (PMC6225804; doi:10.1136/archdischild-2018-314784)
Supplement: Supplementary data [file archdischild-2018-314784supp001.docx]

**Supplementary Table 1: Behaviour change techniques and intervention strategies^12^**

| **Technique^a^** | **Definition^a^** | **Intervention strategies** |
| --- | --- | --- |
| 1. Provide information on consequences | Information about the benefits and costs of action or inaction, focusing on what will happen if the person performs the behaviour. | Leaflet explains link between feeding behaviours, rapid weight gain and risk of obesity.  Information provided during 3 face-to-face and 2 telephone contacts. |
| 2. Prompt intention formation | Encouraging the person to decide to act or set a general goal. | Leaflet encourages lower guidelines for formula-milk feeding and suggests a general feeding plan.  Intervention contacts used to develop a personalised feeding plan (PFP). |
| 3. Prompt barrier identification | Identify barriers to performing the behaviour and plan ways of overcoming them. | Cost-benefit analysis, motivation ruler and confidence ruler.  Formulation of ‘if….then…..’ plans to overcome barriers e.g. crying between feeds (‘If she cries at night, then I will offer her a dummy’) |
| 4. Prompt facilitator identification | Identify facilitators to performing the behaviour and plan ways to use them to overcome barriers. | Cost-benefit analysis, motivation ruler and confidence ruler. |
| 5. Provide general encouragement | Praising or rewarding the person for effort or performance without this being contingent on specified behaviours or standards of performance. | Praise all attempts at following guidelines.  Good communication skills: building rapport, empathy, active listening, non-judgemental, client-centred. |
| 6. Set graded tasks | Set easy task, and increase difficulty until target behaviour is performed. | Monthly contact to encourage mothers to set small achievable goals.  Review of Personal Feeding Plan (PFP) to revise goals. |
| 7. Provide instruction | Telling a person how to perform a behaviour and/or preparatory behaviours. | Two leaflets and discussion about recommended feeding behaviours during 3 face-to-face and 2 telephone contacts. |
| 8. Model or demonstrate the behaviour | An expert shows the person how to correctly perform behaviour eg. in class or on video. | Demonstrate the correct method of formula-feed preparation at baseline visit. |
| 9. Prompt specific goal setting | Involves detailed planning of what the person will do, including a definition of the behaviour specifying frequency, intensity, or duration and specification of at least one context, that is, where, when, how, or with whom. | Personal Feeding plan with goals negotiated with the participant.  Formulation of ‘if….then…..’ plans |
| 10. Prompt review of behavioural goals | Review and/or reconsideration of previously set goals or intentions | Goal review and revised goals set at each intervention contact using the Personal Feeding plan. |
| 11. Prompt self-monitoring | The person is asked to keep a record of specified behaviour(s) (e.g., in a diary). | Participants encouraged to record amount fed in the Personal Feeding plan. |
| 12. Provide feedback on performance | Providing data about recorded behaviour or evaluating performance in relation to a set standard or others’performance, i.e., the person received feedback on their behaviour. | Feedback provided on feeding behaviour, based on Personal Feeding plan. Feedback provided on baby’s growth plotted on growth charts. |
| 13. Teach to use prompts or cues | Teach the person to identify environmental cues that can be used to remind them to perform a behavior,  including times of day or elements of contexts. | Stickers on formula-milk tins encourage lower formula-milk consumption. |

^a^Techniques linked to Social Cognitive Theory and Implementation intentions; and definitions as specified in Abraham and Michie’s Taxonomy of Behaviour Change Techniquess^20^

**Supplementary Table 2: Intervention and Control contacts and content^12^**

| **Timeline** | **Intervention Group (IG)** | **Control Group (CG)** |
| --- | --- | --- |
| 1^st^: Face-to-face.  Within 14 weeks of birth | -Healthy Growth and Nutrition Leaflet.  -Stickers for formula-milk packets/tins with new guideline daily requirements.  -Education about growth charts, rapid weight gain, obesity risk.  -Personal feeding plan (PFP).  -Model feed preparation if necessary. | - Standard Department of Health bottle feeding leaflet.  -General questions about formula-milk feeding, information sources and decisions. |
| 2^nd^: Telephone.  3-4 months (3-6 weeks later) | -Check understanding of key messages.  -Review of PFP and goal setting. | -General questions about sleep and support with caring for baby. |
| 3^rd^ : Face-to-face (IG) /Telephone (CG)  4-5 months (3-6 weeks later) | -Feedback on growth.  -Weaning advice.  -Review of PFP and goal setting. | -General questions about life after the baby’s birth. |
| 4^th^: Telephone.  5-6 months (3-6 weeks later) | -Review of PFP and goal setting. | -General questions about formula-milk changes and weaning |
| 5^th^ : Face-to-face.  6-7 months (3-6 weeks later) | -Feedback on growth.  -Review of PFP and goal setting. | -Standard Department of Health weaning leaflet.  -Questions about experience of taking part in the study and research in general. |

Identification of barriers and facilitator, problem solving, ‘If…then plans’ were used in all contacts. All contacts were underpinned by good communication skills. The motivation ruler and confidence ruler are used for assessment and to prompt identification of barriers and facilitators. The ‘Cost-benefit analysis’ tool was used as required to improve motivation and confidence.

**Supplementary Table 3: Between group differences in infant diet**

|  | **Control** | **Intervention** | **Difference (95% CI)**  **Intervention vs Control** |
| --- | --- | --- | --- |
| **Baseline (age 2 months)** | n=311 | n=331 |  |
| Age, months | 2.3 (0.8) | 2.3 (1.0) |  |
| Milk intake, ml/day | 898.1 (219.7) | 895.9 (217.6) |  |
| Number (%) receiving solid feeds | 6 (1.9%) | 10 (3.0%) |  |
| Average number of solid feeds | 1.5 (0.8) | 1.3 (0.5) |  |
| **Second contact (age 3 months)** | n=321 | n=315 |  |
| Age, months | 3.6 (3.5) | 3.5 (3.5) |  |
| Milk intake, ml/day | 958.3 (198.1) | 834.8 (157.1) | -123.5 (-151.6 to -95.5) |
| Number (%) receiving solid feeds | 17 (5.3%) | 16 (5.1%) |  |
| Average number of solid feeds | 1.4 (0.6) | 1.5 (0.6) | 0.18 (-0.24 to 0.60) |
| **Third contact (age 4 months)** | n=317 | n=307 |  |
| Age, months | 4.2 (0.7) | 4.1 (1.4) |  |
| Milk intake, ml/day | 988.1 (199.5) | 873.0 (152.9) | -115.1 (-143.0 to -87.1) |
| Number (%) receiving solid feeds | 88 (27.8%) | 62 (20.2%) |  |
| Average number of solid feeds | 1.4 (0.6) | 1.5 (0.6) | 0.13 (-0.07 to 0.33) |
| **Fourth contact (age 5 months)** | n=309 | n=308 |  |
| Age, months | 5.1 (0.9) | 5.1 (1.1) |  |
| Milk intake, ml/day | 964.8 (192.4) | 879.1 (143.3) | -85.7 (-112.6 to -58.8) |
| Number (%) receiving solid feeds | 159 (51.5%) | 152 (49.3%) |  |
| Average number of solid feeds | 1.7 (0.8) | 1.8 (0.7) | 0.03 (-0.13 to 0.20) |
| **Age 6 month Follow-up** | n=313 | n=310 |  |
| Age, months | 6.2 (0.4) | 6.1 (0.3) |  |
| Milk intake, ml/day | 895.9 (193.3) | 836.1 (189.2) | -59.7 (-91.1 to -28.3) |
| Number (%) receiving solid feeds | 269 (85.9%) | 256 (82.6%) |  |
| Average number of solid feeds | 2.3 (0.8) | 2.3 (0.8) | -0.02 (-0.16 to 0.11) |
| **Age when solids introduced** | 4.9 (0.8) | 4.9 (0.8) | -0.02 (-0.16 to 0.11) |
| **Age 8 months 4-day diet diary** | n=202 | n=183 |  |
| Energy intake (kcal/day) | 776.4 (141.1) | 770.1 (121.2) | -6.33 (-32.73 to 20.07) |
| Protein intake (kcal/day) | 23.4 (6.1) | 23.3 (5.1) | -0.14 (-1.27 to 0.99) |
| Carbohydrate intake (kcal/day) | 105.6 (19.4) | 105.1 (17.5) | -0.53 (-4.24 to 3.18) |

Mean (SD) for continuous variables, Number (%) for categorical variables, Average number (SD) of solid feeds is summarized for those whose number of solid feeds>0

**Supplementary Table 4: Intervention and control group costs**

|  | **Per intervention participant** | | **Per control participant** |
| --- | --- | --- | --- |
| **Training time costs** |  |  | |
| Five facilitators, 14 hours each | £14 | - | |
| 2 trainers, 14 hours | £9 | - | |
| 1 psychologist trainer, 3 hours | £1 | - | |
| ***Total training*** | ***£22*** | ***£0*** | |
| **Cost of materials** |  |  | |
| Stickers & stationary† | £1 | - | |
| Printing* | £3 | - | |
| ***Total materials*** | ***£4*** | ***£0*** | |
| **Intervention contact**** |  |  | |
| Initial visit (all) | £68 | £68 | |
| 4-6 week telephone contact (all) | £30 | £30 | |
| 4-5 month Visit (Int) | £64 | - | |
| 4-5 month Call (Cont) | - | £30 | |
| 5-6 month Call (all) | £30 | £30 | |
| 6-7 month visit (all) | £59 | £59 | |
| ***Total intervention contact*** | ***£252*** | ***£218*** | |
| **Intervention non-contact**** |  |  | |
| Travel costs home visits‡ | £23 | £23 | |
| Missed calls/appointments | £22 | £19 | |
| ***Total costs per participant*** | ***£323*** | ***£260****˄* | |

†project receipts; *estimate from UEA print shop; ‡project expenses ** Mean reported duration from Time diaries, costed from PSSRU Health visitor rate

**Supplementary Table 5: Health service resource use (non-routine) and costs in intervention and control groups**

| Mean number of contacts up to age 12 months | **Intervention** (n=269) | | **Control** (n=277) | | **Cost comparison** |
| --- | --- | --- | --- | --- | --- |
|  | Mean number of contacts | SD | Mean number of contacts | SD | Mean Difference costs (95% CI) |
| GP | 2.53 | 2.36 | 3.12 | 2.93 | -£26 (-46, -7) |
| Health visitor | 0.78 | 2.15 | 0.65 | 1.75 | £7 (-11, 25) |
| Practice nurse | 0.20 | 0.94 | 0.18 | 0.64 | £0 (-2, 2) |
| Outpatient | 0.39 | 0.98 | 0.31 | 0.73 | £16 (-13, 46) |
| Hospital admission/day case | 0.41 | 0.07 | 0.30 | 0.06 | £69 (-46, 184) |
| A&E | 0.28 | 0.65 | 0.28 | 0.59 | £3 (-20, 26) |
| All other healthcare contacts | 0.23 | 0.87 | 0.25 | 0.05 | -£1 (-7, 7) |
|  |  |  |  |  |  |
| **Health resource use (HRU) costs** | **£571** | **953** | **£504** | **786** | **£67 (-80, 213)** |
| **Total cost*** | **£894** | **953** | **£764** | **786** | **£130 (-25, 268)** |

*Health resource use + Intervention costs

**Supplementary Table 6: Summary of effective behavioural intervention trials- Blake-Lamb 2016 Systematic review^9^**

| **Trial** | **Reference** | **Intervention target** | **Intervention duration** | **Outcome (intervention vs control)** |
| --- | --- | --- | --- | --- |
| STRIP | Hakenen 2006 | Family diet & physical activity | Birth to 10yrs | At age 10 fewer girls overweight |
| SLIMTIME | Paul 2011 | Child sleep & diet-responsive feeding | 2wks to 6mo | Lower weight for length centile |
| Healthy Beginnings | Wen 2012 | Family-child diet & physical activity-responsive feeding | Pregnancy to 2yrs | Lower BMI |
| Lifestyle Counselling | Mustilla 2012 | Mother diet & physical activity | 2mo to 4yrs | Slower increase in BMI z-score |
| Prevention of overweight | Verbesteel 2013 | Family diet & physical activity | 9mo to 3yrs | Greater decrease in BMI z-score |
| Maternal-child Pastoral | Navarro 2013 | Mother-child diet, parenting | Pregnancy to 2yrs | Lower BMI z-score |
| NOURISH | Daniels 2013 | Child diet & physical activity-responsive feeding | 4mo to 15mo | Lower BMI z-score at 13-15mo, no difference at 2yrs |
