# Supplementary figures and images for: Randomised controlled trial of a theory-based behavioural intervention to reduce formula milk intake
Source: Arch Dis Child. 2018 May 14;103(11):1054–60. doi: 10.1136/archdischild-2018-314784 (PMC6225804; doi:10.1136/archdischild-2018-314784)

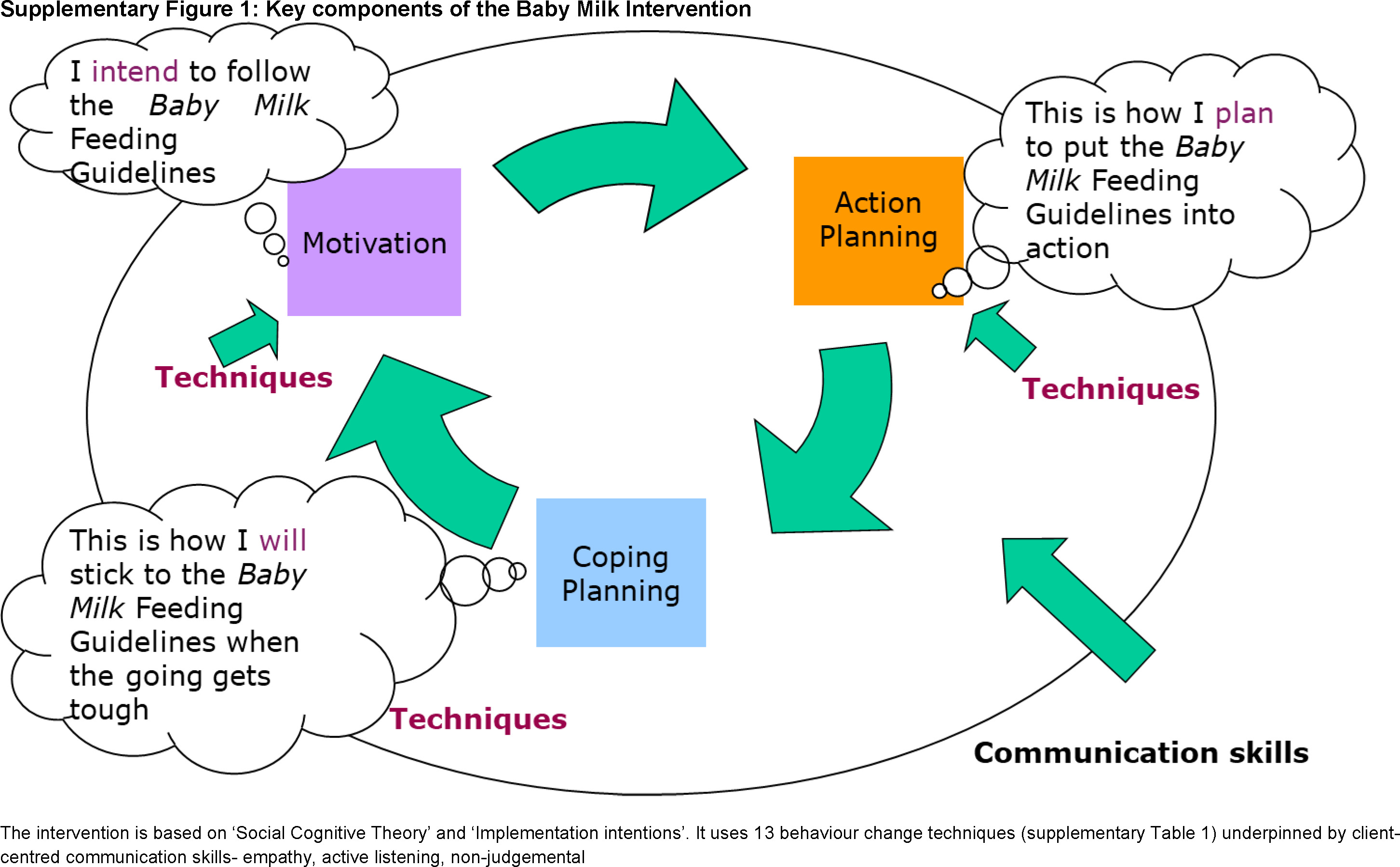

Supplement: Supplementary data [file archdischild-2018-314784supp002.jpg]

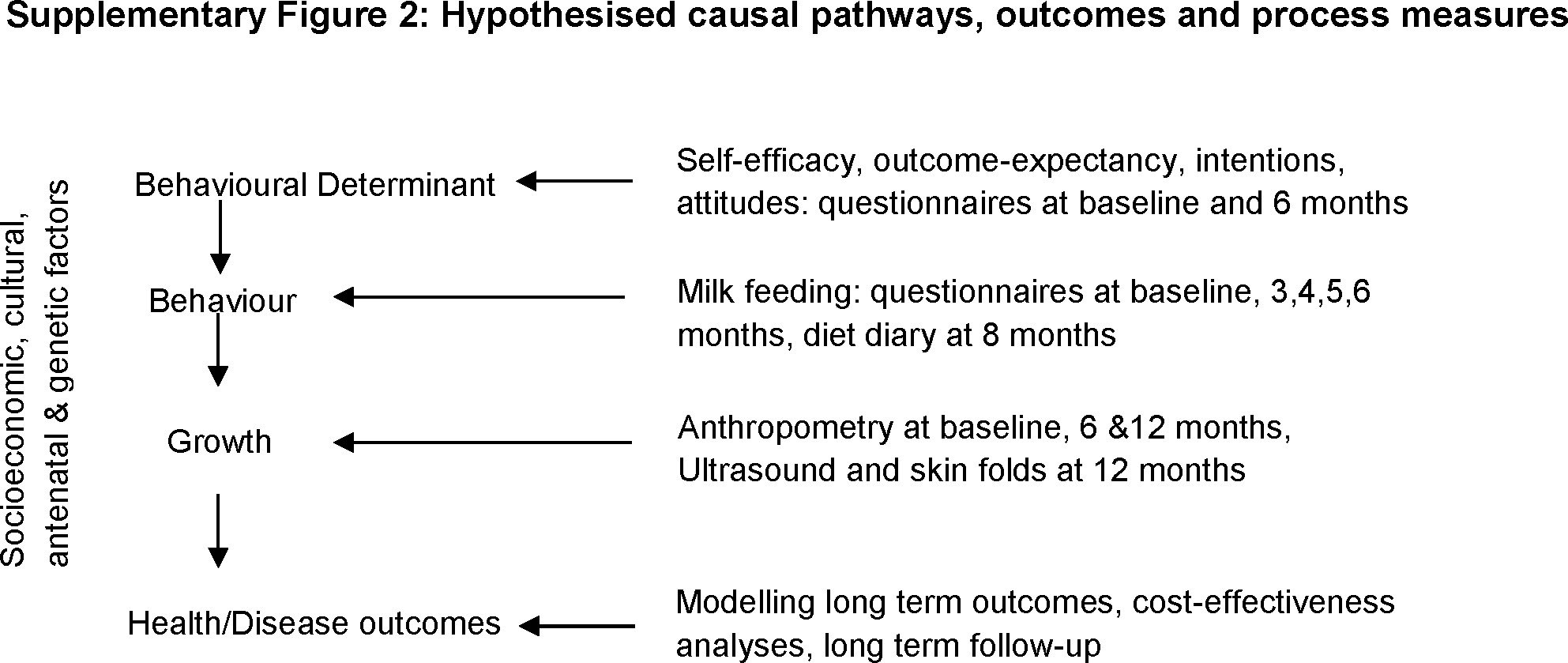

Supplement: Supplementary data [file archdischild-2018-314784supp003.jpg]

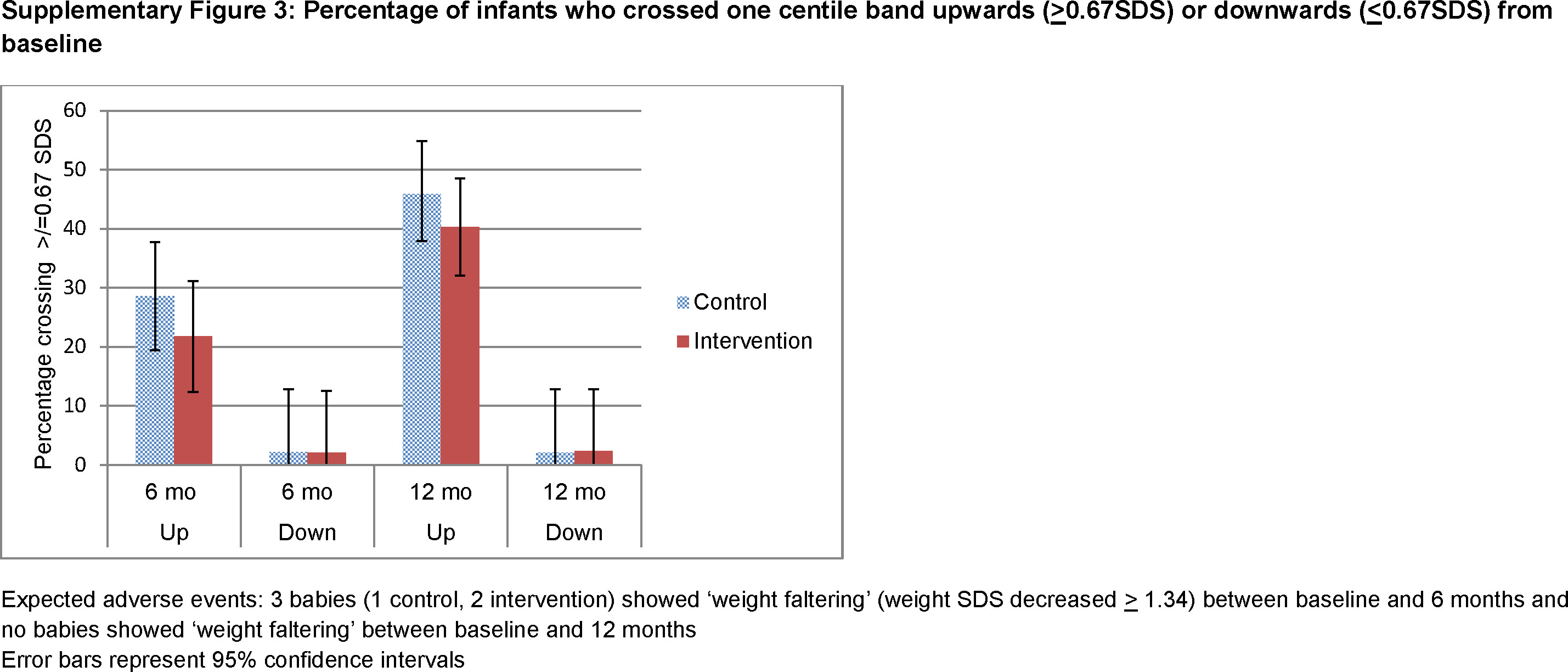

Supplement: Supplementary data [file archdischild-2018-314784supp004.jpg]

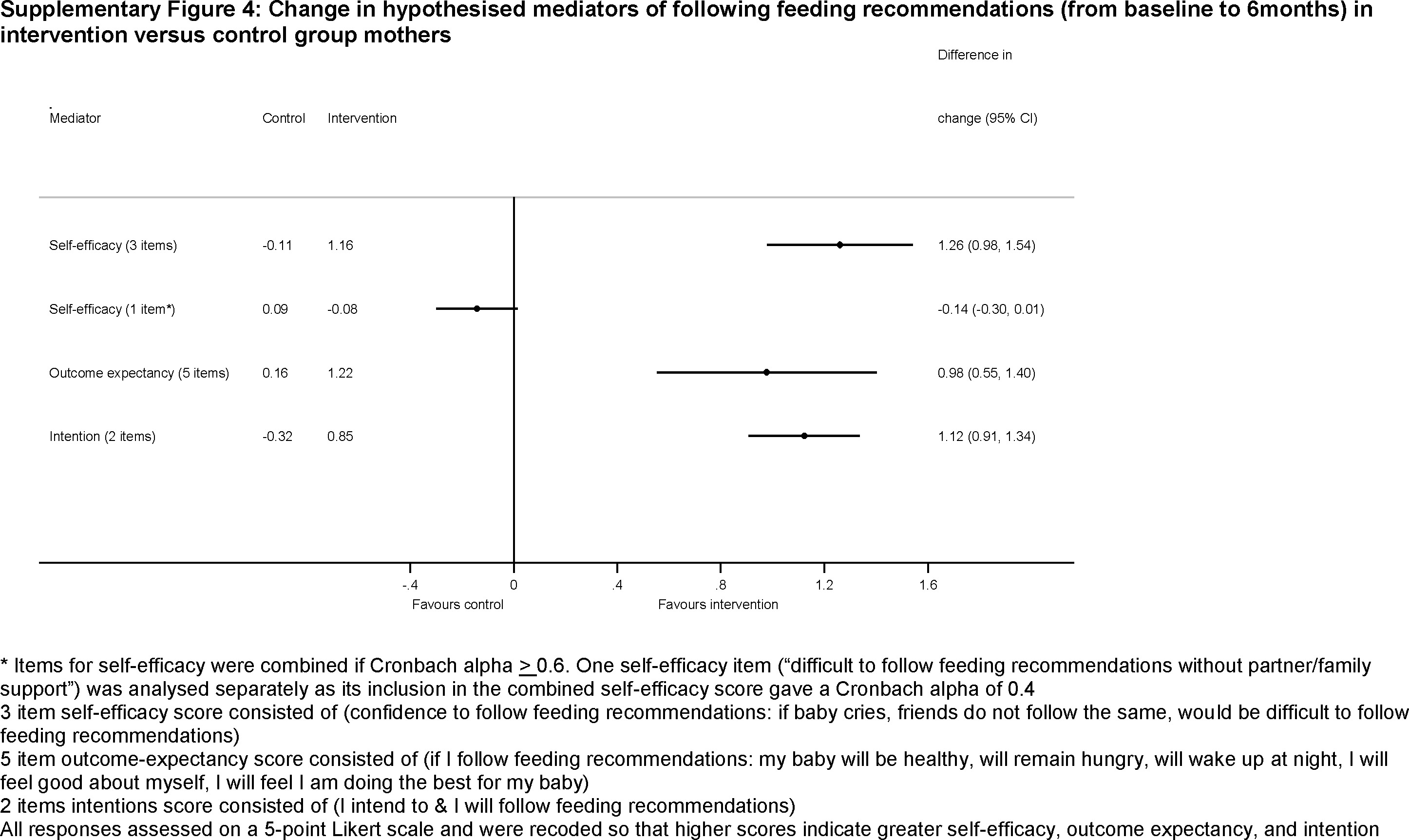

Supplement: Supplementary data [file archdischild-2018-314784supp005.jpg]

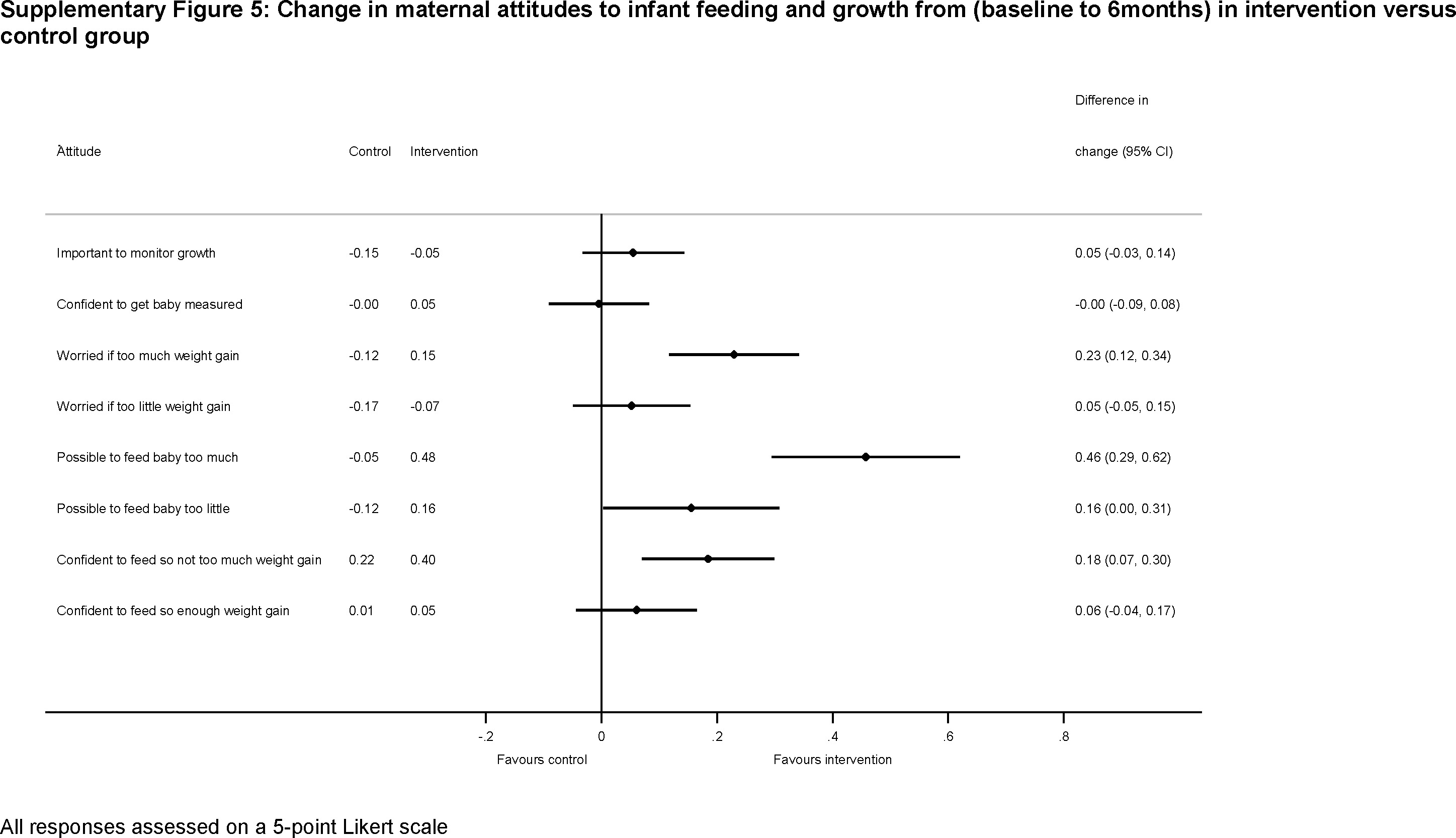

Supplement: Supplementary data [file archdischild-2018-314784supp006.jpg]
